# Neurological Sequelae After Paediatric Cryptococcal Meningitis

**DOI:** 10.3390/jof11110767

**Published:** 2025-10-24

**Authors:** Alison Gifford, Simran Atulkumar Patel, Masilo Matlakala, Rachael Dangarembizi, Adilia Warris

**Affiliations:** 1 Medical Research Council Centre for Medical Mycology at the University of Exeter, Department of Biosciences, Faculty of Health and Life Sciences, Geoffrey Pope Building, Stocker Road, Exeter EX4 4QD, UK; a.warris@exeter.ac.uk; 2 CMM AFRICA Medical Mycology Research Unit, Institute of Infectious Diseases and Molecular Medicine, University of Cape Town, Cape Town 7701, South Africa; ptlsim004@myuct.ac.za (S.A.P.); mtlmas031@myuct.ac.za (M.M.); rachael.dangarembizi@uct.ac.za (R.D.); 3 Department of Human Biology, University of Cape Town, Cape Town 7701, South Africa

**Keywords:** paediatric, HIV, *Cryptococcus neoformans*, *Cryptococcus gattii*, cryptococcal meningitis, neurodevelopment, visual impairment

## Abstract

An infectious insult to a child’s developing brain has the potential to result in life-long neurological and neurodevelopmental consequences. Adult survivors of cryptococcal meningitis (CM) can suffer from long-term neurological sequelae such as blindness and motor weakness, but little is known about outcomes in children. A PubMed and Ovid Global Health search identified all children <19 yrs of age with proven cryptococcal disease of the central nervous system until October 2024. A total of 868 children were included from 108 publications. In total, 555 (67%) were HIV positive, 67 (8%) non-HIV immunocompromised and 204 (25%) immunocompetent. The mortality rate was 24% (104/430). No child had a documented formal neurodevelopmental assessment after CM. Of those with a documented clinical outcome, 20% (36/184) had neurological sequelae, but this was higher in HIV-positive children (25%, 11/44). Visual impairment was most commonly observed (13%, 23/184) and remarkably higher in those with *Cryptococcus gattii* meningitis (32%, 10/31). Other sequelae included limb weakness (*n* = 8), learning difficulties (*n* = 7), hearing loss (*n* = 3) and recurrent seizures (*n* = 2). The burden of neurological sequelae is likely even more extensive than captured, with little data available from the populations most affected by CM. It is vital that neurodevelopmental assessment of children after CM is standard in all countries to support rehabilitation and the best functional outcomes.

## 1. Introduction

An infectious insult to a child’s developing brain has the potential to result in life-long neurological and neurodevelopmental consequences as it intersects with vulnerable phases of brain development [1]. This can include not only gross- and fine-motor, hearing and vision, but also have profound impacts on the child’s cognition, behaviour and schooling.

Adult survivors of cryptococcal meningitis (CM) often suffer from long-term neurological sequelae such as visual impairment, hearing loss, cognitive impairment, limb weakness and seizure disorders [2,3,4,5]. We have recently published an extensive review of 1469 paediatric cases with cryptococcal disease [6]. As in adults, it predominantly affects children living with HIV who comprised 65% of cases, and the majority (54.2%) of children lived in Africa. The overall mortality was 23%, but higher (32%) in children living with HIV [6]. However, very little is known about the neurological and neurodevelopmental outcomes in the children who survive CM.

Neurological sequelae in children due to pneumococcal meningitis include focal deficits (3–14%), hearing loss (14–25%), seizures (15–63%) and cognitive impairment (4–41%) [7]. Extensive studies have also been performed to elucidate outcomes after tuberculous meningitis (TBM) in children, with 54% of survivors having neurological sequelae including motor deficits (7–48%), speech (14–33%), hearing loss (6–19%), visual impairment (3–44%) and seizures (3–27%) [8].

Due to this high burden of post-meningitis long-term sequelae, some countries such as the United Kingdom advise a review with a paediatrician 4–6 weeks after discharge for a meningitis-related admission to assess hearing and the presence of neurological and neurodevelopmental problems [9]. In South Africa, which has the highest incidences of CM overall [10], there are extensive guidelines for the management of acute meningitis in children [11] and follow-up is recommended. However, only children under one year of age qualify for neurodevelopmental follow-up, and audiology testing is only recommended after confirmed bacterial meningitis [12,13].

It is important that long-term neurodevelopmental problems are identified early to make prompt referrals to rehabilitation therapies and education to maximise a child’s potential after meningitis. For example, tailored physiotherapy and occupational therapy will leverage neuroplasticity and increase muscle strength, balance and the ability to complete functional activities to improve a child’s quality of life and independence. In addition, a procedure such as cochlear implantation for post-meningitis deafness is more effective the sooner it is performed after the acute event [14].

We hypothesise that there is a significant risk of neurological sequelae after CM in the developing brains of children that justifies neurodevelopmental assessment in survivors. This study aims to characterise the long-term neurological burden caused by CM through a literature review of paediatric cases.

## 2. Methods

Our previously published review of paediatric cryptococcosis served as a starting point, which contained clinical data of 1048 children from the 143 publications included in the data analysis. The PubMed and Ovid Global Health database search was updated by searching for the keywords (“cryptococcosis” OR “cryptococcal meningitis” OR “*Cryptococcus neoformans*” OR “*Cryptococcus gattii*”) AND (CNS OR meningitis OR “central nervous system”) to cover the period from January 2023 to October 2024. The same inclusion criteria were applied as the original review [6], with only studies published in the English language and with children <19 years of age included. To be included in the present data analysis, cases needed to have proven cryptococcal disease of the CNS. CNS cryptococcal disease was defined as a positive India ink stain, cryptococcal antigen (CrAg) test, culture or polymerase chain reaction on cerebrospinal fluid (CSF), or brain imaging suggestive of CNS disease with a positive blood culture for *Cryptococcus* sp. and a contraindication for lumbar puncture. In addition, a case had to have information about four of the following seven characteristics: sex, underlying disease, signs and symptoms, diagnostic test results, species identification, treatment and outcome. For this review, data extracted from old and new papers included mortality, neurological and neurodevelopmental assessment at discharge and/or follow-up, and the presence and timing of follow-up. We analysed the data as HIV positive if >90% of the children were HIV positive and as non-HIV if <20% of the children were diagnosed with HIV in a study population, when the results were presented for the total population only.

Data analysis was performed using R version 4.2.2 (R Foundation for Statistical Computing, Vienna, Austria). Countries were grouped according to World Bank classifications [15]. The Kruskal–Wallis, chi-squared or Fisher’s exact test was used to compare groups with odds ratio (OR) and 95% confidence interval (CI) shown where appropriate.

## 3. Results

### 3.1. Demographics

**Figure 1:**
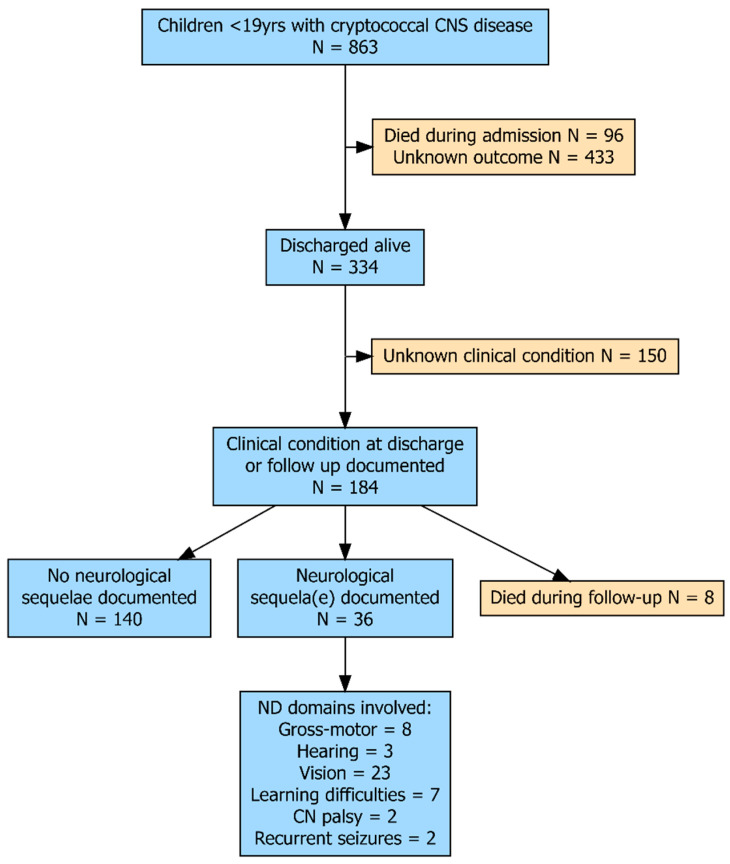
Flow diagram of children included in the review. CNS: central nervous system; CN = cranial nerve.

One hundred and eight publications were found describing cryptococcal disease of the CNS in children, including 101 studies from the previous paediatric cryptococcosis review and seven from the updated database search (see references in Supplementary File S1). A total of 863 children aged 0–18 years were included (see Figure 1). Of those with a documented sex (*n* = 411), 61% were boys. Median age was difficult to quantify as in many of the larger studies children of similar age were grouped together. Only including studies describing individual children (*n* = 72), the median age was 9 years (interquartile range 5–13 years).

The majority of children with cryptococcal meningitis came from the African continent and were living with HIV (see Table 1). There were 107 children from high-income countries (HIC), 748 from middle-income countries (MIC) and only 8 from low-income countries (LIC) in this review. There were 37 children for whom the underlying condition was not known [16,17]. For the remaining 826 children, the underlying conditions were as follows: 67% (555/826) were living with HIV, 8% (67/826) were immunocompromised for reasons other than HIV and 25% (204/826) were documented as immunocompetent. For cases in which the *Cryptococcus* species was known, 89% (464/522) were *C. neoformans*, 10% (54/522) were *C. gattii* and 1% (4/522) were others including *C. laurentii*., *C. albidus* and *C. humicolis*. Of the 51 cases of *C. gattii* meningitis where the underlying diagnosis was known, 53% (27/51) were immunocompetent, 29% (15/51) were HIV positive and 18% (9/51) were non-HIV immunocompromised.

### 3.2. Mortality

Of the children with a known outcome, the inpatient mortality rate was 22% (96/430). There was no significant difference in mortality between HIC, MIC and LIC (23% [21/92] vs. 22% [74/332] vs. 17% [1/6]) but it was higher in children living with HIV than non-HIV immunocompromised and immunocompetent children (28% [49/172] vs. 22% [14/65] vs. 17% [33/192]; *p* = 0.04). A further eight children died during follow-up (five non-HIV immunocompromised children and three HIV-positive children), equating to an overall mortality of 24% (104/430).

### 3.3. Follow-Up After Discharge

In total, 50% (168/334) of inpatient survivors had a follow-up after discharge documented. The median duration of follow-up was 12 months (IQR 6–24 months). This was longer for non-HIV immunocompromised children (18 months [IQR 12–29]) than immunocompetent children (12 months [IQR 6–12]) and HIV-positive children (8 months [IQR 4–61]), but not statistically significantly (*p* = 0.06). This was also the case for follow-up duration in HIC (18 months [IQR 12–32] compared to MIC (9 months [IQR 6–19]) and LIC (6 months [IQR 4–9]; *p* = 0.07).

### 3.4. Neurological or Neurodevelopmental Assessment

No patient had a documented formal neurodevelopmental assessment at discharge or follow-up. In total, 55% (184/334) of patients who were discharged alive had documented data about their clinical condition at discharge or during follow-up. Mostly, this was very brief such as “showed good clinical response”. Significantly less children living with HIV had a documented clinical condition than non-HIV immunocompromised or immunocompetent children (36% [44/123] vs. 80% [41/51] vs. 62% [99/159]; *p* < 0.001).

Of those with a documented clinical outcome, 20% (36/184) had documented neurological sequelae [18,19,20,21,22,23,24,25,26,27,28,29,30,31,32,33,34,35,36,37,38,39,40] (see Supplementary File S2). This includes around a quarter of HIV-positive children (25% [11/44]) and immunocompetent children (23% [23/99]). In contrast, only 5% (2/41) of non-HIV compromised children had neurological sequelae documented (*p* = 0.01).

### 3.5. Vision

The most common neurological complication was visual impairment, with 13% (23/184) of children with a documented clinical condition at discharge or follow-up having blindness or reduced visual acuity after treatment for CM. This included five children from HIC and 18 from MIC with a median age of 11.5 years (IQR 9–13) and 68% male. Vision was the only neurological complication for 17 of the 23 patients, and the remaining six children also had gross-motor sequelae, learning difficulties or hearing loss.

**Figure 2:**
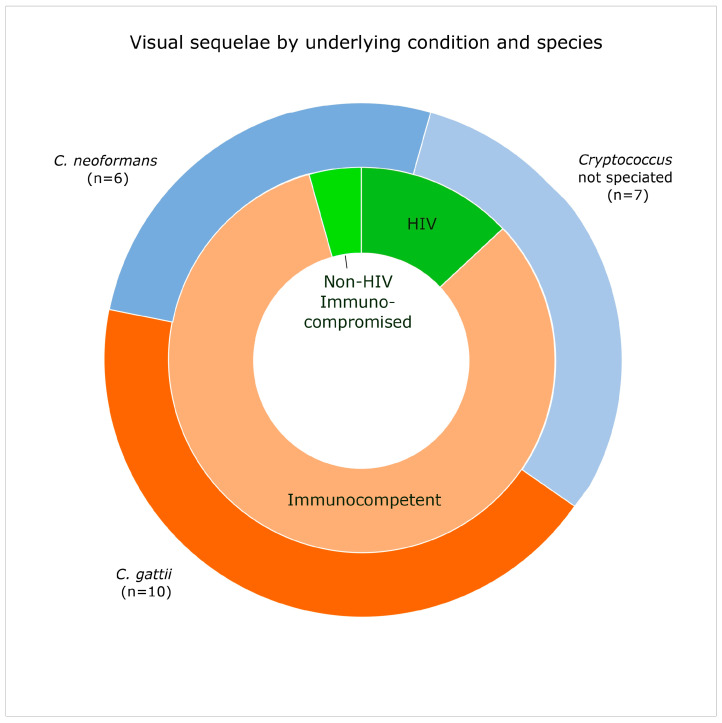
Doughnut chart representing the 23 children with visual sequelae after cryptococcal meningitis by underlying condition and *Cryptococcus* species.

Visual complications were more often associated with a *C. gattii* infection (*n* = 10) than *C. neoformans* (*n* = 6; see Figure 2), meaning that 32% (10/31) of children with *C. gattii* meningitis who had a documented clinical outcome had visual impairment, compared to 5% (6/113) of those with *C. neoformans* meningitis (*p* < 0.001; OR 8, 95% CI 2–31). All 10 children with *C. gattii* meningitis and visual impairment were immunocompetent. Nine other immunocompetent children had visual impairment (four with *C. neoformans* and five unspeciated) whilst only three HIV-positive children (one *C. neoformans* and two unspeciated) and one non-HIV immunocompromised children (*C. neoformans*) were affected. There were significantly more immunocompetent children with visual sequelae than non-HIV immunocompromised children (19% [19/99] vs. 2% [1/41]; *p* = 0.007) but not HIV-positive children (7% [3/44]).

In 9 of the 23 patients, visual symptoms were present at admission including diplopia and “blindness”. However, for some patients the visual symptoms began during inpatient treatment, for example, bilateral acute visual loss on day 18 [22] or day 42 [19] of treatment. Visual complications were highly associated with raised intracranial pressure (ICP). ICP was raised in all children with visual impairments who had a documented ICP (*n* = 17) and nine underwent a CSF shunt procedure. One child had partial resolution of visual impairment by discharge [20], but otherwise visual complications present at discharge appeared to be long-standing and documented up to five-year follow-up in one study [30]. Of note, only one study had a documented measurement of visual acuity (6/18) for an 8-year-old boy in India [19].

### 3.6. Motor Weakness

Limb weakness, predominantly bilateral lower limb weakness, was present at discharge in 4% (8/184) of children, one from HIC and seven from MIC, with a median age of 7.5 years (IQR 5.5–11). This includes a two-month-old HIV-exposed but HIV PCR-negative baby who was believed to have acquired CM due to vertical transmission [23]. He was discharged at nine months of age with global developmental delay. Also included is the only non-meningitis CNS case in this review is a 12-year-old immunocompetent female with cryptococcal spinal arachnoiditis who had ongoing weakness and spasticity of her lower limbs at eight months post-discharge, but this was reported to be improving [25]. In four children the limb weakness was starting to improve at the time of discharge.

There is no documentation of any of these children, nor any of the other 863 children in this review, having a more detailed gross-motor neurodevelopmental examination at either discharge or follow-up. Additionally, there is no documentation of a fine-motor skills examination for any child after CM.

### 3.7. Learning Difficulties

Including two children documented as having “psychomotor retardation”, 4% (7/184) of children had documented learning difficulties after CM. In three children, the learning difficulties were present more than six years after CM [27]. No assessment methods or more detailed description of their difficulties were given for any child. The median age was 10 years (IQR 10–13); five were children living with HIV and two were immunocompetent. One child was from HIC and six from MIC and all with a documented sex were male. Speciation was known for only two children who had *C. neoformans* meningitis. ICP when measured (*n* = 5) was raised in all five children. Only two children had learning difficulties as the sole neurological sequalae, association with blindness, motor difficulties and/or hearing loss was present in the others.

### 3.8. Other Neurological Outcomes

Hearing loss was documented in two girls and one boy at discharge, two immunocompetent children and one non-HIV immunocompromised child with ages ranging from 8 to 15 years. An eight-year-old girl from Malawi underwent a formal hearing test showing mild to moderate sensorineural hearing loss unilaterally that was improving at two-month follow-up [21]. In another child the hearing impairment had recovered by the six-month follow-up [35].

Two immunocompetent children had recurrent seizures after discharge. One five-year-old boy from Brazil had *C. gattii* CM and had lower limb hypotonus and blindness as well as seizures [22]. The second child, from China, also had numerous other neurological sequelae [33].

One immunocompetent ten-year-old boy from Papua New Guinea left the hospital after *C. gattii* CM with an ongoing bilateral cranial nerve VI palsy and no documented follow-up [39]. One seven-year-old HIV-positive male from South Africa developed cranial nerve VI and VII palsy one month after discharge with an MRI finding of Acute Disseminated EncephaloMyelitis (ADEM) [34].

## 4. Discussion

This is the largest review of neurological outcomes of CM in children on a global scale. One in five children (20%) with a documented clinical outcome had neurological sequelae in at least one domain including vision, motor, learning difficulties, hearing or recurrent seizures. Around one in four HIV positive and immunocompetent children had neurological sequelae. The most common neurological outcome was visual impairment affecting 13% of children with a documented clinical outcome, increasing to 32% in those with *C. gattii* meningitis.

Visual impairment is a major complication in adults with CM, with 19% of adults having profound visual loss assessed by visual acuity [41] and in up to 52% of those with *C. gattii* meningitis [42]. It is known that visual impairment is more common after *C. gattii* meningitis than *C. neoformans* meningitis [43], but the pathophysiology is incompletely understood. It is thought to be due to either direct fungal invasion of the optic nerve, localised arachnoiditis or raised ICP, and has been shown to be independently associated with raised ICP and fungal burden [44]. It is possible that the larger inflammatory response seen in immunocompetent individuals leads to increased damage to the optic nerve. Aggressive management of raised ICP by repeated lumbar punctures or CSF shunt procedures is thought to help prevent visual loss and recommended in international management guidelines [45]. Our main finding of visual impairment after *C. gattii* meningitis explains the high rate of neurological sequelae seen in immunocompetent children in this review and reflects the immunocompetent population from HIC and MIC.

Overall, the most important finding of this review is how little is known about neurological or neurodevelopmental outcomes after paediatric CM. There is no documentation of a formal neurodevelopmental examination for any child in the medical literature. There is no documented follow-up for 50% of children who survived admission, therefore their long-term outcomes are not known. The majority of documented outcomes are “blindness” or lower limb weakness, which are likely to have been easily evident at discharge without any additional testing. More “subtle” sequelae that require additional examination such as fine-motor impairment were not documented. Therefore, the burden of sequelae is likely far more extensive than captured in the reviewed literature. In addition, we have assumed that any brief documentation of a “good clinical response” equates to no neurological sequelae, when it is possible that some of these children did not have a full neurological examination at discharge and so sequelae were missed.

There is a very low percentage of children with HIV in Africa with a documented clinical follow-up in the literature. This is, in part, because some of the larger studies in this population do not focus on clinical outcomes [46]. However, this is a vulnerable group due to comorbidities and, although small numbers, this review shows that one quarter of HIV-positive children with documentation of clinical outcome have sequelae. Children with longer follow-up periods show that neurological sequelae, such as learning difficulties, are present many years after the infectious insult [27]. It is likely that areas of the world with later and more severe presentations to hospital will have more significant neurological sequelae. In one-third (204/555) of HIV-positive children with CM, the *Cryptococcus* species was not known. When the species was known, 95% of cases were caused by *C. neoformans,* suggesting that *C. neoformans* may be less likely to cause visual sequelae than *C. gattii*, but these children are still at significant risk of visual and other sequelae. More data are urgently needed from this population.

It is unclear why neurological sequelae were significantly less frequent in those who were non-HIV immunocompromised. They have similar proportions of *C. neoformans* and *C. gattii* to the immunocompetent group. Five children from this group did pass away during follow-up. A high proportion were from HIC, which perhaps meant earlier access to medical care and gold-standard therapy, not always available to children in resource-limited settings.

A prospective study using validated, setting-appropriate neurodevelopmental testing in a high-burden area would help more accurately understand how CM affects all domains of neurodevelopment and compare the duration and severity of neurological sequelae to those in adults. A cost-effective analysis of rehabilitation therapies against disability-adjusted life-years would help advocate for the correct testing and therapies to be put in place. The National Institute for Health and Care Excellence (NICE) in the United Kingdom considers it cost effective to follow-up and assess children for neurological complications for at least two years after bacterial meningitis to mitigate health and educational harm [47].

In conclusion, with evidence of neurological sequelae in 1 in 5 children after cryptococcal meningitis, it is vital that neurodevelopmental assessment including vision and hearing is placed in national guidelines to support rehabilitation and the best functional outcomes. In addition, this review provides further evidence to argue for improved recognition, diagnostics and management of cryptococcal meningitis in the paediatric cohort to lessen the neurological impact of this disease.

## Figures and Tables

**Table 1:** Children with cryptococcal central nervous system disease shown by underlying diagnosis.

	Total (*N* = 863) ^†^	HIV (*N* = 555)	Non-HIV Immunocompromised (*N* = 67)	Immuno- Competent (*N* = 204)	*p* *
Males	61% (250/411)	60% (102/170)	48% (24/50)	66% (109/166)	n.s
Age in years (IQR)	9 (5–13)	9.5 (8–10)	12.5 (8–15)	8 (3–12)	n.s
Continent:					
Africa	57% (494/862)	88% (489/554)	0%	2% (5/204)	
Asia	22% (193/862)	4% (21/554)	34% (23/67)	73% (148/204)	
Europe	1% (7/862)	<1% (1/554)	7% (5/67)	<1% (1/204)	
North America	8% (68/862)	6% (34/554)	37% (25/67)	4% (9/204)	
South America	9% (80/862)	2% (9/554)	12% (8/67)	13% (27/204)	
Oceania	2% (20/862)	0%	9% (6/67)	7% (14/204)	
*Cryptococcus* species:					<0.001
*neoformans*	89% (464/522)	95% (333/351)	80% (41/51)	76% (84/111)	
*gattii*	10% (54/522)	4% (15/351)	18% (9/51)	24% (27/111)	
other ^#^	1% (4/522)	1% (3/351)	2% (1/51)	0%	
Inpatient mortality	22% (96/430)	28% (49/172)	22% (14/65)	17% (33/192)	0.04
General follow-up (% of survivors)	50% (168/334)	28% (34/123)	57% (29/51)	66% (105/159)	<0.001
Duration follow-up months (IQR)	12 (6–24)	8 (4–61)	18 (12–29)	12 (6–12)	n.s
Death during follow-up	2% (8/334)	2% (3/123)	10% (5/51)	0% (0/159)	<0.001
Any documented clinical condition at discharge or follow-up	55% (184/334)	36% (44/123)	80% (41/51)	62% (99/159)	<0.001
Neurological sequelae	20% (36/184)	25% (11/44)	5% (2/41)	23% (23/99)	0.01
Domains involved:					
Gross-motor	4% (8/184)	5% (2/44)	2% (1/41)	5% (5/99)	n.s
Hearing	2% (3/184)	0	2% (1/41)	2% (2/99)	n.s
Vision	13% (23/184)	7% (3/44)	2% (1/41)	19% (19/99)	0.01
Learning difficulties	4% (7/184)	11% (5/44)	2% (1/41)	1% (1/99)	0.01
CN palsy	1% (2/184)	2% (1/44)	0	1% (1/99)	n.s
Recurrent seizures	1% (2/184)	0	0	2% (2/99)	n.s

^†^ Includes the 37 children for whom the underlying condition was not known. * Pearson χ^2^ test; Fisher exact test, Kruskal–Wallis test. Performed among the 3 groups: HIV positive, immunocompetent and non-HIV immunocompromised. ^#^ Including *Cryptococcus laurentii*, *Cryptococcus albidus* and *Cryptococcus humicolis*. CN = cranial nerve; HIV = human immunodeficiency virus; IQR = interquartile range; n.s = not significant.

## Data Availability

The original contributions presented in this study are included in the article/Supplementary Material. Further inquiries can be directed to the corresponding author.

## Supplementary Materials

The following supporting information can be downloaded at https://www.mdpi.com/article/10.3390/jof11110767/s1; File S1: Reference list of 108 studies included in the review. File S2: Table of papers with neurological sequelae.
