# Supplementary material for: Neurological Sequelae After Paediatric Cryptococcal Meningitis
Source: J Fungi (Basel). 2025 Oct 24;11(11):767. doi: 10.3390/jof11110767 (PMC12653805; doi:10.3390/jof11110767)
Supplement: Supplementary file 1 [file jof-11-00767-s001.zip › Supp File S1 Reference list.pdf]

Case reports

1. Belokda T, Hamadi H, Ait M'Barek Y, et al. Subacute Hydrocephalus Revealing a Cryptococcus Infection in a Seven-Year-Old Child. Curēus. 2024;16(3):e56372.
2. Barut D, Kunay B, Yıldırım Arslan S, et al. Disseminated cryptococcosis in a child with liver transplantation: a case report. Turkish Journal Pediatrics. 2024;66(4):499-504.
3. Huang J, Li H, Lan C, et al. Disseminated cryptococcal infection with pulmonary involvement presenting as diffuse cavitary nodules in an immunocompromised patient: a case report. BMC Pulmonay Medicine. 2023;23(1):38.
4. Firacative C, Zuluaga-Puerto N, Guevara J. Cryptococcus neoformans Causing Meningoencephalitis in Adults and a Child from Lima, Peru: Genotypic Diversity and Antifungal Susceptibility. Journal of Fungi (Basel). 2022;8(12).
5. Mesquita AL, Coutinho JVSC, Ferreira Filho LA, et al. Cranial base pachymeningitis in children: beyond tuberculosis. Pediatric Infectious Disease Journal. 2022;41(4):e175-e7.
6. Miller C, Daugherty R, McCulloch M, et al. Immune reconstitution inflammatory syndrome complicating cryptococcal meningitis in a pediatric heart transplant patient. Pediatric Infectious Disease Journal. 2022;41(2):145-7.
7. Dhoubhadel BG, Laghu U, Poudel R, et al. A rare case of cryptococcal meningitis in a child with a congenital heart disease. Case Reports in Infectious Diseases. 2021;2021.
8. Pallavi S, Meeta S, Tanu S, et al. Cryptococcus neoformans in a child with CSF rhinorrhoea: a cytopathological diagnosis. Diagnostic Cytopathology. 2021;49(9):E348-E51.
9. Song Q, Guan Y. A case of acute pancreatitis induced by voriconazole during treatment of cryptococcal meningitis. British Journal of Clinical Pharmacology. 2021;88(4):1925-9.
10. Cheng JH, Cheema R, Williamson PR, et al. Case Report: Paradoxical Inflammatory Response Syndrome in a Previously Healthy, HIV-Negative, Pediatric Patient With Cryptococcus gatii Meningitis. Frontiers in Pediatrics. 2021;9.
11. Nakatudde I, Kasirye P, Kiguli S, et al. It is not always tuberculosis! A case of pulmonary cryptococcosis in an immunocompetent child in Uganda. African Health Sciences. 2021;21(3):990-4.
12. Athipongarporn A, Ittiwut C, Manuyakorn W, et al. Diagnosis of hyper IgM syndrome in a previously healthy adolescent boy presented with cutaneous and cerebral cryptococcosis. Pediatric Infectious Disease Journal. 2021;40(1):e18-e20.
13. Zhang N, Chen K, Zhu JS, et al. Sequential infection of Epstein-Barr virus and cryptococcal encephalitis after umbilical cord blood transplantation in a child with X-linked adrenoleukodystrophy. Pediatric Transplantation. 2021;25(5):e13956.
14. Huynh J, Saddi V, Cooper P, et al. Unusual Presentation of Severe Endobronchial Obstruction Caused by Cryptococcus gattii in a Child. Journal of the Pediatric Infectious Diseases Society. 2020;9(1):67-70.
15. Otto SBJ, George PE, Mercedes R, et al. Cryptococcal meningitis and immune reconstitution inflammatory syndrome in a pediatric patient with HIV after switching to second line antiretroviral therapy: a case report. BMC Infectious Diseases. 2020;20(68).
16. Berejnoi A, Taverna CG, Mazza M, et al. First case report of cryptococcosis due to Cryptococcus decagattii in a pediatric patient in Argentina. Revista da Sociedade Brasileira de Medicina Tropical. 2019;52(12).
17. Cotton MF, Rabie H, Nemes E, et al. A prospective study of the immune reconstitution inflammatory syndrome (IRIS) in HIV-infected children from high prevalence countries. PLoS one. 2019;14(7).

18. Suzuki SML, Morelli F, Negri M, et al. FATAL cryptococcal meningitis in a child with hyper-immunoglobulin M syndrome, with an emphasis on the agent. Journal de Mycologie Medicale. 2019;29(3):273-7.
19. Padmanabha H, Kasinathan A, Kumar A, et al. Vision Loss in an 8-Year-Old Immunocompetent Boy with Cryptococcal Meningitis. The Pediatric Infectious Disease Journal. 2018;37(8).
20. Kocabas B, Emin Parlak M, Özhak Baysan B, et al. Disseminated Cryptococcosis With Severe Increased Intracranial Pressure Complicated With Cranial Nerve Palsy in a Child. The Pediatric Infectious Disease Journal. 2018;37(4):373-5.
21. Javed I, Muthu C, Jhuma S, et al. Disseminated cryptococcosis presenting as miliary lung shadows in an immunocompetent child. Journal of Tropical Pediatrics. 2018;64(5):434-7.
22. Mohanty SK, Deepshi T, Devika G, et al. Diminished CD40L expression on T-cells in a case of disseminated cryptococcosis. Indian Journal of Pathology & Microbiology. 2018;61(1):137-40.
23. Chimowa T, King I, Tam P, et al. Cryptococcal meningitis in a previously healthy child. Malawi Medical Journal. 2017;29(4):330-1.
24. O'Reilly DA. A rare case of neonatal cryptococcal meningitis in an HIV-unexposed 2-day-old infant: the youngest to date? Paediatrics and International Child Health. 2016;36(2):154-6.
25. Shukla S, Garg J, Mahajan G, et al. Liver dysfunction as the presenting feature of disseminated cryptococcosis. Southern African Journal of Infectious Diseases. 2016;31(2):38-40.
26. Kaur H, Zaman K, Thapa BR, et al. Fatal cryptococcosis involving multiple sites in an immunocompetent child. Indian Journal of Medical Microbiology. 2015;33(5 (Suppl):148-50.
27. Martinez-Longoria CA, Rubio-Perez NE, Rios-Solis JE, et al. Severe meningoencephalitis co-infection due to Cryptococcus neoformans and Mycobacterium tuberculosis in a child with systemic lupus erythematosus. International Journal of Infectious Diseases. 2015;33:106-8.
28. Valente ES, Lazzarin MC, Koech BL, et al. Disseminated cryptococcosis presenting as cutaneous cellulitis in an adolescent with systemic lupus erythematosus. Infectious Disease Reports. 2015;7(2):22-4.
29. Alsum Z, Al-Saud B, Al-Ghonaium A, et al. Disseminated cryptococcal infection in patient with novel JAK3 mutation severe combined immunodeficiency, with resolution after stem cell transplantation. Pediatric Infectious Disease Journal. 2012;31(2):204-6.
30. Heath JL, Yin DE, Wechsler DS, et al. Successful treatment of disseminated cryptococcal infection in a pediatric acute lymphoblastic leukemia patient during induction. Journal of Pediatric Hematology and Oncology. 2012;34(4):e161-e3.
31. Natukunda E, Musiime V, Ssali F, et al. A case of cryptococcal lymphadenitis in an HIV-infected child. AIDS Research and Human Retroviruses. 2011;27(4):373-6.
32. Nagotkar L, Shanbag P, Mauskar A, et al. Fulminant intracranial hypertension due to cryptococcal meningitis in a child with nephrotic syndrome. Indian Journal of Critical Care Medicine. 2011;15(3):176-8.
33. Pinto Junior VL, Pone MVdS, Pone SM, et al. Cryptococcus gattii molecular type VGII as agent of meningitis in a healthy child in Rio de Janeiro, Brazil: report of an autochthonous case. Revista da Sociedade Brasileira de Medicina Tropical. 2010;43(6):746-8.

34. Liou J, Chiu C, Tseng C, et al. Cryptococcal meningitis in pediatric systemic lupus erythematosus. Mycoses. 2003;46(3-4):153-6.
35. Nakwan N, Ngerncham S, Srisuparp P, et al. Cryptococcus neoformans septicemia in an immunocompetent neonate: first case report in Thailand. The Southeast Asian Journal of Tropical Medicine and Public Health. 2008;39(4):697-700.
36. Shen Y, Wang J, Lu H. Voriconazole in an infant with cryptococcal meningitis. Chinese Medical Journal. 2008;121(3):286-8.
37. Swe KS, Bekker A, Greeff S, et al. Cryptococcus meningitis and skin lesions in an HIV negative child. Journal of Clinical Pathology. 2008;61(10):1138-9.
38. Yilmaz-Demirdag Y, Wilson B, Lowery-Nordberg M, et al. Interleukin-2 treatment for persistent cryptococcal meningitis in a child with idiopathic CD4+ T lymphocytopenia. Allergy and Asthma Proceedings. 2008;29(4):421-4.
39. van Toorn R, Rabie H. Pseudocystic cryptococcal meningitis complicated by transient periaqueductal obstruction in a child with HIV infection. European Journal of Paediatric Neurology. 2005;9(2):81-4.
40. Kumari PR, Shahapur PR, Rao PS. Corticosteroid induced Cryptococcus meningitis. Indian Journal of Medical Microbiology. 2005;23(3):207-8.
41. Simon G, Simon G, Erdo"os M, et al. Invasive Cryptococcus laurentii disease in a nine-year-old boy with X-linked hyper-immunoglobulin M syndrome. Pediatric Infectious Disease Journal. 2005;24(10):935-7.
42. Jenney A, Pandithage K, Fisher DA, et al. Cryptococcus infection in tropical Australia. Journal of Clinical Microbiology. 2004;42(8):3865-8.
43. Pasqualotto AC, Bittencourt Severo C, de Mattos Oliveira F, et al. Cryptococcemia. An analysis of 28 cases with emphasis on the clinical outcome and its etiologic agent. Revista Iberoamericana de Micología. 2004;21(3):143-6.
44. Ching N, Lasky J, Lazareff J, et al. Enlarging parietal mass with lytic skull lesion. Pediatric Infectious Disease Journal; 2004. 2004;23(6):589.
45. Mavinkurve-Groothuis AM, Bokkerink JP, Verweij PE, et al. Cryptococcal meningitis in a child with acute lymphoblastic leukemia. Pediatric Infectious Disease Journal. 2003;22(6):576.
46. Sirinavin S, Intusoma U, Tuntirungsee S. Mother-to-child transmission of Cryptococcus neoformans. Pediatric Infectious Disease Journal. 2004;23(3):278-9.
47. Kaur R, Mittal N, Rawat D, et al. Cryptococcal meningitis in a neonate. Scandinavian Journal of Infectious Diseases. 2002;34(7):542-3.
48. Tuerlinckx D, Bodart E, Garrino MG, et al. Cutaneous lesions of disseminated cryptococcosis as the presenting manifestation of human immunodeficiency virus infection in a twenty-two-month-old child. Pediatric Infectious Disease Journal. 2001;20(4):463-4.
49. Urbini B, Castellini C, Rondelli R, et al. Cryptococcal meningitis during front-line chemotherapy for acute lymphoblastic leukemia. Haematologica. 2000;85(10):1103-4.
50. Buckingham SC, San Joaquin VH. Cerebral cryptococcomas in an adolescent with X-linked agammaglobulinemia. Infectious Diseases in Clinical Practice. 1999;8(5):262-6.
51. Ruggieri M, Polizzi A, Vitaliti MC, et al. Fatal biphasic brainstem and spinal leptomeningitis with Cryptococcus neoformans in a non-immunocompromised child. Acta Paediatrica. 1999;88(6):671-4.
52. Numata K, Tsutsumi H, Wakai S, et al. A child case of haemophagocytic syndrome associated with cryptococcal meningoencephalitis. Journal of Infection. 1998;36(1):118-9.

53. Mirdha BR, Sarkar T, Banerjee U, et al. Concurrent cryptococcal meningitis and falciparum malaria in a child with nephrotic syndrome. Indian Pediatrics. 1998;35(4):360-3.
54. Miniero R, Nesi F, Vai S, et al. Cryptococcal meningitis following a thrombotic microangiopathy in an unrelated donor bone marrow transplant recipient. Pediatric Hematology and Oncology. 1997;14(5):469-74.
55. Laverda AM, Ruga E, Pagliaro A, et al. Intracranial hypertension and cryptococcal meningitis in a girl with AIDS. Brain & Development. 1996;18(4):330-1.
56. Schoeman JF, Honey EM, Loock DB. Raised ICP in a child with cryptococcal meningitis: CT evidence of a distal CSF block. Child's Nervous System. 1996;12(9):568-71.
57. Chen C, Wang K. Cryptococcal meningitis in pregnancy. American Journal of Perinatology. 1996;13(1):35-6.
58. Iseki M, Anzo M, Yamashita N, et al. Hyper-IgM immunodeficiency with disseminated cryptococcosis. Acta Paediatrica. 1994;83(7):780-2.
59. Arisoy ES, Arisoy AE, Dunne WM, Jr. Clinical significance of fungi isolated from cerebrospinal fluid in children. Pediatric Infectious Disease Journal. 1994;13(2):128-33.
60. Ting SF, Glader BE, Prober CG. Cryptococcus infection in a nine-year-old child with hemophilia and the acquired immunodeficiency syndrome. Pediatric Infectious Disease Journal. 1991;10(1):76.
61. Al-Rasheed SA, Al-Fawaz IM. Cryptococcal meningitis in a child with systemic lupus erythematosus. Annals of Tropical Paediatrics. 1990;10(3):323-6.
62. Moncino MD, Gutman LT. Severe systemic cryptococcal disease in a child: review of prognostic indicators predicting treatment failure and an approach to maintenance therapy with oral fluconazole. Pediatric Infectious Disease Journal. 1990;9(5):363-8.
63. Woodall WC, III, Bertorini TE, Bakhtian BJ, et al. Spinal arachnoiditis with Cryptococcus neoformans in a nonimmunocompromised child. Pediatric neurology. 1990;6(3):206-8.
64. Harper KJ, Sawyer WT. Malabsorption of flucytosine in a pediatric patient with Shwachman syndrome. DICP, the Annals of Pharmacotherapy. 1989;23(10):782-3.
65. Reinig JW, Hungerford GD, Mohrmann ME, et al. Case report 268. Skeletal Radiology. 1984;11(3):221-3.
66. Oteyza EN. Cryptococcal meningitis in a 1 month old infant. Acta Medica Philippina. 1981;17(1):24-7.

Case series and larger studies

1. Petrikkos L, Kourti M, Antoniadi K, et al. Central Nervous System Fungal Diseases in Children with Malignancies: A 16-Year Study from the Infection Working Group of the Hellenic Society of Pediatric Hematology Oncology. Journal of Fungi. 2024;10(9):654.
2. Puplampu P, Asamoah I, Asare BO, et al. Cryptococcal meningitis among perinatally HIV-infected adolescents: Case series on presentation and management challenges. Clinical Case Reports. 2023;11(2):e6995.
3. Vidal JE, Oliveira FG, Vieira M, et al. Finger-Prick Whole Blood Cryptococcal Antigen Lateral Flow Assay for the Diagnosis of Cryptococcosis in HIV-Negative Patients: A Case Series Study in Two Tertiary Centers in São Paulo, Brazil. Journal of Fungi (Basel). 2023;9(12).
4. Kaur H, Gupta P, Pilania R, et al. Trend of pediatric cryptococcosis in a tertiary care centre and review of literature. Indian Journal of Medical Microbiology. 2023;43:18-29.
5. Enicker B, Aldous C. Cerebrospinal Fluid Shunting in Children with Hydrocephalus and Increased Intracranial Pressure Secondary to Human Immunodeficiency Virus-Related Cryptococcal Meningitis. World Neurosurgery. 2022;168:e530-e7.
6. Pacharn P, Phongsamart W, Boonyawat B, et al. Disseminated cryptococcosis in two boys with novel mutation of CD40 Ligand-Associated X-linked hyper-IgM syndrome. Asian Pacific Journal of Allergy and Immunology. 2021;39(1):31-4.
7. Huang J, Liu C, Zheng X. Clinical features of invasive fungal disease in children with no underlying disease. Scientific Reports. 2022;12(1).
8. Bouille JGd, Epelboin L, Henaff F, et al. Invasive cryptococcosis in French Guiana: immune and genetic investigation in six non-HIV patients. Frontiers in Immunology. 2022;13(April).
9. Nguefack S, Taguebue J, Wandji Y, et al. Neuromeningeal cryptococcosis in children: clinical and prognostic aspects in a pediatric hospital in Yaounde - Cameroon. Pediatric OnCall. 2020;17(3):77-81.
10. Hong N, Chen M, Xu N, et al. Genotypic diversity and antifungal susceptibility of Cryptococcus neoformans isolates from paediatric patients in China. Mycoses. 2019;62(2):171-80.
11. Grimshaw A, Palasanthiran P, Huynh J, et al. Cryptococcal infections in children: retrospective study and review from Australia. Future Microbiology. 2019;14:1531-44.
12. O'Brien MP, Ford TJ, Currie BJ, et al. Cryptococcus gattii infection complicated by immune reconstitution inflammatory syndrome in three apparently immunocompetent children. Journal of Paediatrics and Child Health. 2019;55(8):943-7.
13. Gao L, Jiao A, Wu X, et al. Clinical characteristics of disseminated cryptococcosis in previously healthy children in China. BMC Infectious Diseases. 2017;17(359).
14. Nyazika TK, Masanganise F, Hagen F, et al. Cryptococcal Meningitis Presenting as a Complication in HIV-infected Children: A Case Series From Sub-Saharan Africa. Pediatric Infectious Disease Journal. 2016;35(9):979-80.
15. Dou H-T, Xu Y-C, Wang H-Z, et al. Molecular epidemiology of Cryptococcus neoformans and Cryptococcus gattii in China between 2007 and 2013 using multilocus sequence typing and the DiversiLab system. European Journal of Clinical Microbiology & Infectious Diseases. 2015;34(4):753-62.
16. Luo F, Tao Y, Wang Y, et al. Clinical study of 23 pediatric patients with cryptococcosis. European Review for Medical and Pharmacological Sciences. 2015;19(20):3801-10.

17. Hassan H, Cotton MF, Rabie H. Complicated and protracted cryptococcal disease in HIV-infected children. Pediatric Infectious Disease Journal. 2015;34(1):62-5.
18. Lizarazo J, Escandón P, Agudelo CI, et al. Cryptococcosis in Colombian children and literature review. Memórias do Instituto Oswaldo Cruz. 2014;109:797-804.
19. Guo J, Zhou J, Zhang S, et al. A case-control study of risk factors for HIV-negative children with cryptococcal meningitis in Shi Jiazhuang, China. BMC Infectious Diseases. 2012;12:376.
20. Meiring ST, Quan VC, Cohen C, et al. A comparison of cases of paediatric-onset and adult-onset cryptococcosis detected through population-based surveillance, 2005–2007. AIDS. 2012;26(18):2307-14.
21. Miglia KJ, Govender NP, Rossouw J, et al. Analyses of pediatric isolates of Cryptococcus neoformans from South Africa. Journal of Clinical Microbiology. 2011;49(1):307-14.
22. Ramdial PK, Sing Y, Deonarain J, et al. Pediatric renal cryptococcosis: novel manifestations in the acquired immunodeficiency syndrome era. International Journal of Surgical Pathology. 2011;19(3):386-92.
23. Yuanjie Z, Jianghan C, Nan X, et al. Cryptococcal meningitis in immunocompetent children. Mycoses. 2012;55(2):168-71.
24. Huang K, Huang Y, Hung I, et al. Cryptococcosis in nonhuman immunodeficiency virus-infected children. Pediatric Neurology. 2010;42(4):267-70.
25. Jiang P-F, Yu H-M, Zhou B-L, et al. The role of an Ommaya reservoir in the management of children with cryptococcal meningitis. Clinical Neurology and Neurosurgery. 2010;112(2):157-9.
26. Joshi NS, Fisher BT, Prasad PA, et al. Epidemiology of cryptococcal infection in hospitalized children. Pediatric Infectious Disease Journal. 2010;29(12):e91-e5.
27. Severo CB, Xavier MO, Gazzoni AF, et al. Cryptococcosis in children. Paediatric respiratory reviews. 2009;10(4):166-71.
28. Puthanakit T, Oberdorfer P, Akarathum N, et al. Immune reconstitution syndrome after highly active antiretroviral therapy in human immunodeficiency virus-infected Thai children. Pediatric Infectious Disease Journal. 2006;25(1):53.
29. Likasitwattanakul S, Poneprasert B, Sirisanthana V. Cryptococcosis in HIV-infected children. The Southeast Asian Journal of Tropical Medicine and Public Health. 2004;35(4):935-9.
30. Kaur R, Rawat D, Kakkar M, et al. Cryptococcal meningitis in pediatric AIDS. Journal of Tropical Pediatrics. 2003;49(2):124-5.
31. Banerjee U, Datta K, Casadevall A. Serotype distribution of Cryptococcus neoformans in patients in a tertiary care center in India. Medical Mycology. 2004;42(2):181-6.
32. Correa MdPSC, Severo LC, Oliveira FdM, et al. The spectrum of computerized tomography (CT) findings in central nervous system (CNS) infection due to Cryptococcus neoformans var. gattii in immunocompetent children. Revista do Instituto de Medicina Tropical de São Paulo. 2002;44:283-7.
33. Gumbo T, Kadzirange G, Mielke J, et al. Cryptococcus neoformans meningoencephalitis in African children with acquired immunodeficiency syndrome. Pediatric Infectious Disease Journal. 2002;21(1):54-6.
34. Abadi J, Nachman S, Kressel AB, et al. Cryptococcosis in children with AIDS. Clinical Infectious Disease. 1999;28(2):309-13.

35. Subramanyam VR, Mtitimila E, Hart CA, et al. Cryptococcal meningitis in African children. Annals of Tropical Paediatrics. 1997;17(2):165-7.
36. Laurenson I, Trevett A, Lalloo D, et al. Meningitis caused by Cryptococcus neoformans var. gattii and var. neoformans in Papua New Guinea. Transactions of the Royal Society of Tropical Medicine and Hygiene. 1996;90(1):57-60.
37. Marwaha RK, Trehan A, Jayashree K, et al. Hypereosinophilia in disseminated cryptococcal disease. Pediatric Infectious Disease Journal. 1995;14(12):1102-3.
38. Rozenbaum R, Gonçalves AJR. Clinical epidemiological study of 171 cases of cryptococcosis. Clinical Infectious Disease. 1994;18(3):369-80.
39. Fisher D, Burrow J, Lo D, et al. Cryptococcus neoformans in tropical northern Australia: predominantly variant gattii with good outcomes. Australian and New Zealand Journal of Medicine. 1993;23(6):678-82.
40. Leggiadro RJ, Barrett FF, Hughes WT. Extrapulmonary cryptococcosis in immunocompromised infants and children. Pediatric Infectious Disease Journal. 1992;11(1):43-7.
41. Leggiadro RJ, Kline MW, Hughes WT. Extrapulmonary cryptococcosis in children with acquired immunodeficiency syndrome. Pediatric Infectious Disease Journal. 1991;10(9):658-62.
42. Bateson EM. Computed tomography of intracranial torulosis in the Australian aboriginal. Australasian Radiology. 1986;30(2):92-5.
