# Supplementary material for: Neurological Sequelae After Paediatric Cryptococcal Meningitis
Source: J Fungi (Basel). 2025 Oct 24;11(11):767. doi: 10.3390/jof11110767 (PMC12653805; doi:10.3390/jof11110767)
Supplement: Supplementary file 1 [file jof-11-00767-s001.zip › Supp File S2 Table of papers with neurological sequelae.pdf]

Ref	No. children with CNS involvement	Age (yrs) (median or range)	Sex	Underlying condition	Country	*Cryptococcus* species	Survival	General follow-up	Follow-up period	Neurodevelopmental sequelae
[1]	1	12	M	Immunocompetent	Brazil	*C. gattii*	Y	Y	1 year	Lower-limb weakness and visual acuity deficit
[2]	1	8	M	Immunocompetent	India	*C. neoformans*	Y	Y	3 months	Visual acuity is 6/18 in both eyes.
[3]	1	12	F	Immunocompetent	Turkey	*C. neoformans*	Y	Y	1 year	Partial visual loss
[4]	1	8	F	Immunocompetent	Malawi	*Cryptococcus* species	Y	Y	2 months	Mild-moderate unilateral sensorineural hearing loss improving at 2 months
[5]	1	5	M	Immunocompetent	Brazil	*C. gattii*	Y	Y	-	Hypotonus, seizures, bilateral blindness
[6]	1	0	M	Prematurity	Thailand	*C. neoformans*	Y	Y	-	Globally delayed development
[7]	1	12	F	Immunocompetent	South Africa	*Cryptococcus species*	Y	N	-	Bilateral optic atrophy and blindness
[8]	1	12	F	Immunocompetent	USA	*C. neoformans*	Y	Y	8 months	Spinal arachnoiditis with leg weakness and spasticity at 8 months, although improving.
[9]	12	-	-	Mixed	India	*C. neoformans*	1 with disseminated infection died	N	-	Quadriparesis.
[10]	17	10	13 male; 4 female	HIV	South Africa	*Cryptococcus* species	3 died	Y	1 – 128 months	Visual impairment (2), learning difficulties (4), paraparesis (2)
[11]	3	4 – 17	3 male	Mixed	French Guiana	*C. gattii* and *C. neoformans*	100% survival	N	-	Psychomotor retardation, hearing loss and blindness in one child.
[12]	12	11	5 male; 7 female	HIV	Cameroon	*C. neoformans*	7 died	N	-	Blindness (1) and psychomotor retardation (1)
[13]	6	6 – 15	4 male; 2 female	Mixed	Australia	*C. gattii* and *C. neoformans*	1 died	Y	14 months to 5 years	Visual loss (1)

[14]	3	8 – 13	3 male	Immunocompetent	Australia	*C. gattii*	100% survival	Y	2 years	No neurological sequelae (2), severe residual visual impairment secondary to optic atrophy (1)
[15]	36	1 – 11	27 male; 9 female	Immunocompetent	China	*Cryptococcus species*	6 died	Y	-	Blindness.
[16]	19*	-	-	Immunocompetent	China	*Cryptococcus* species	At least 5 with CNS disease died	Y	>6 months	One case suffered paralysis, seizures, mental retardation, aphasia, blindness, and other complications
[17]	3	7 – 10	2 male; 1 female	HIV	South Africa	*C. neoformans*	100% survival	Y	12 months	CN palsy 1 month after initiating ART (MRI showed ADEM) and raised ICP 6–7 months later.
[18]	23	0 – 17	14 male; 9 female	Mixed	China	*C. neoformans*	2 died	Y	6 months	At discharge: hearing damage (1) and diplopia (1). Recovered by follow-up.
[19]	11	2 – 15	7 male, 4 female	Immunocompetent	China	*Cryptococcus* species	2 died	Y	2 years	Visual loss (1)
[20]	9	9 – 18	2 male; 7 female	Mixed	Taiwan	*C. neoformans*	100% survival	N	-	Optic dystrophy and blindness (1)
[21]	11	6 –12	8 male; 3 female	Immunocompetent	Brazil	*C. gattii*	2 died	Y	-	Blindness (5)
[22]	5	8 – 18	3 male; 2 female	Immuno-competent	Papua New Guinea	*C. neoformans and C. gattii*	1 died	N	-	Bilateral 6th CN palsy and visual loss (1)
[23]	3	7 – 16	1 male; 2 female	Mixed	Australia	*Cryptococcus species*	1 died	Y	2 weeks	Visual loss (1)

Supplementary Table S1: Studies which include children with neurological sequelae after cryptococcal CNS disease.

CNS = central nervous system; CN = cranial nerve; ART = antiretroviral therapy; MRI = magnetic resonance imaging; ADEM = acute demyelinating encephalomyelitis; ICP = intracranial pressure

* In Kaur *et al* 2023 and Luo *et al* 2015, some of the clinical details for those with CNS disease cannot be separated from those with pulmonary disease without CNS disease.

1. Mesquita AL, Coutinho JVSC, Ferreira Filho LA, et al. Cranial base pachymeningitis in children: beyond tuberculosis. Pediatric Infectious Disease Journal. 2022;41(4):e175-e7.
2. Padmanabha H, Kasinathan A, Kumar A, et al. Vision Loss in an 8-Year-Old Immunocompetent Boy with Cryptococcal Meningitis. The Pediatric Infectious Disease Journal. 2018;37(8).
3. Kocabas B, Emin Parlak M, Özhak Baysan B, et al. Disseminated Cryptococcosis With Severe Increased Intracranial Pressure Complicated With Cranial Nerve Palsy in a Child. The Pediatric Infectious Disease Journal. 2018;37(4):373-5.
4. Chimowa T, King I, Tam P, et al. Cryptococcal meningitis in a previously healthy child. Malawi Medical Journal. 2017;29(4):330-1.
5. Pinto Junior VL, Pone MVdS, Pone SM, et al. Cryptococcus gattii molecular type VGII as agent of meningitis in a healthy child in Rio de Janeiro, Brazil: report of an autochthonous case. Revista da Sociedade Brasileira de Medicina Tropical. 2010;43(6):746-8.
6. Sirinavin S, Intusoma U, Tuntirungsee S. Mother-to-child transmission of Cryptococcus neoformans. Pediatric Infectious Disease Journal. 2004;23(3):278-9.
7. Schoeman JF, Honey EM, Loock DB. Raised ICP in a child with cryptococcal meningitis: CT evidence of a distal CSF block. Child's Nervous System. 1996;12(9):568-71.
8. Woodall WC, III, Bertorini TE, Bakhtian BJ, et al. Spinal arachnoiditis with Cryptococcus neoformans in a nonimmunocompromised child. Pediatric neurology. 1990;6(3):206-8.
9. Kaur H, Gupta P, Pilania R, et al. Trend of pediatric cryptococcosis in a tertiary care centre and review of literature. Indian Journal of Medical Microbiology. 2023;43:18-29.
10. Enicker B, Aldous C. Cerebrospinal Fluid Shunting in Children with Hydrocephalus and Increased Intracranial Pressure Secondary to Human Immunodeficiency Virus-Related Cryptococcal Meningitis. World neurosurgery. 2022;168:e530-e7.
11. Bouille JGd, Epelboin L, Henaff F, et al. Invasive cryptococcosis in French Guiana: immune and genetic investigation in six non-HIV patients. Frontiers in Immunology. 2022;13(April).
12. Nguefack S, Taguebue J, Wandji Y, et al. Neuromeningeal cryptococcosis in children: clinical and prognostic aspects in a pediatric hospital in Yaounde - Cameroon. Pediatric OnCall. 2020;17(3):77-81.
13. Grimshaw A, Palasanthiran P, Huynh J, et al. Cryptococcal infections in children: retrospective study and review from Australia. Future Microbiology. 2019;14:1531-44.
14. O'Brien MP, Ford TJ, Currie BJ, et al. Cryptococcus gattii infection complicated by immune reconstitution inflammatory syndrome in three apparently immunocompetent children. Journal of Paediatrics and Child Health. 2019;55(8):943-7.
15. Gao L, Jiao A, Wu X, et al. Clinical characteristics of disseminated cryptococcosis in previously healthy children in China. BMC infectious diseases. 2017;17(359).

16. Luo F, Tao Y, Wang Y, et al. Clinical study of 23 pediatric patients with cryptococcosis. European Review for Medical and Pharmacological Sciences. 2015;19(20):3801-10.
17. Hassan H, Cotton MF, Rabie H. Complicated and protracted cryptococcal disease in HIV-infected children. Pediatric Infectious Disease Journal. 2015;34(1):62-5.
18. Guo J, Zhou J, Zhang S, et al. A case-control study of risk factors for HIV-negative children with cryptococcal meningitis in Shi Jiazhuang, China. BMC infectious diseases. 2012;12:376.
19. Yuanjie Z, Jianghan C, Nan X, et al. Cryptococcal meningitis in immunocompetent children. Mycoses. 2012;55(2):168-71.
20. Huang K, Huang Y, Hung I, et al. Cryptococcosis in nonhuman immunodeficiency virus-infected children. Pediatric neurology. 2010;42(4):267-70.
21. Correa MdPSC, Severo LC, Oliveira FdM, et al. The spectrum of computerized tomography (CT) findings in central nervous system (CNS) infection due to Cryptococcus neoformans var. gattii in immunocompetent children. Revista do Instituto de Medicina Tropical de São Paulo. 2002;44:283-7.
22. Laurenson I, Trevett A, Lalloo D, et al. Meningitis caused by Cryptococcus neoformans var. gattii and var. neoformans in Papua New Guinea. Transactions of the Royal Society of Tropical Medicine and Hygiene. 1996;90(1):57-60.
23. Bateson EM. Computed tomography of intracranial torulosis in the Australian aboriginal. Australasian radiology. 1986;30(2):92-5.
